# A study on rapid simulation of mine roadway fires for emergency decision-making

**DOI:** 10.1038/s41598-024-51900-3

**Published:** 2024-01-19

**Authors:** Yangqin Chen, Jian Liu, Qichao Zhou, Li Liu, Dong Wang

**Affiliations:** 1 College of Safety Science and Engineering, Liaoning Technical University, Huludao, 125105 Liaoning China; 2 Key Laboratory of Mine Thermo-Motive Disaster and Prevention, Ministry of Education, Huludao, 125105 Liaoning China; 3 Software (Artificial Intelligence) Institute, Liaoning Technical University, Huludao, 125105 Liaoning China; 4 Liaoning Academy of Mineral Resources Development and Utilization Technical and Equipment Research Institute, Liaoning Technical University, Fuxin, 123000 China

**Keywords:** Energy science and technology, Engineering

## Abstract

In traditional mine fire simulation, the FDS simulation software has been verified by large-scale and full-size fire experiments. The resulting calculations closely align with real-world scenarios, making it a valuable tool for simulating mine fires. However, when a fire occurs in a mine, utilizing FDS software to predict the fire situation in the mine entails a sequence of steps, including modeling, environmental parameter setting, arithmetic, and data processing, which takes time in terms of days, thus making it difficult to meet the demand for emergency decision-making timelines. To address the need for rapid predictions of mine tunnel fire development, a method for swiftly estimating environmental parameters and the concentration of causative factors at various times and locations post-fire has been devised. FDS software was employed to simulate numerous roadway fires under diverse conditions. Parameters such as fire source intensity, roadway cross-sectional area, roadway wind speed, roadway inclination angle, time, and others were utilized as the input layer for a neural network. In contrast, wind flow temperature, carbon monicide (CO) concentration, fire wind pressure, visibility, and others were designated as the output layer for training the neural network model. This approach established a fire prediction model to resolve issues related to time-consuming numerical simulations and the inability to provide a rapid response to disaster emergencies. The trained neural network model can instantaneously predict the environmental parameters and concentrations of the causative factors at different times and locations. The model exhibits an average relative error of 12.12% in temperature prediction, a mean absolute error of 0.87 m for visibility, a mean absolute error of 3.49 ppm for CO concentration, and a mean absolute error of 16.78 Pa for fire wind pressure. Additionally, the mean relative error in density is 2.9%. These predictions serve as crucial references for mine fire emergency decision-making.

## Introduction

When a mine fire occurs, it poses a significant threat to underground workers, equipment, localized production systems, and even the whole mine production system. This threat arises from the unique operating conditions within mines, such as restricted spaces in mines and the combustion by-products. Therefore, the rapid and accurate prediction of environmental parameters and factors causing disasters at various mine locations during a fire is of great significance to the safety of mine production. Previous researchers have conducted extensive research on roadway fires using various approaches, including theoretical analysis^1–11^, numerical simulation^12–19^, and experimental studies on roadway fires^20–24^. With the rapid advances in computational capabilities, computational fluid dynamics (CFD) has emerged as a significant method for simulating real fires. Various commercial software tools, such as fluent, and FDS, have been developed for this purpose. FDS primarily simulates the flow movement in fires, using numerical methods to solve the Navier–Stokes (N–S) equations governing buoyancy-driven flows at low Mach numbers. These simulations have been validated through large-scale, full-size fire experiments, yielding results that closely align with real-world scenarios. In previous studies, W. Budryk proposed a preliminary theory regarding local fire wind pressure and the concept of smoke excess. He theoretically analyzed the phenomena related to wind flow reversal, smoke flow reversal, and retreat in mine fires^2^. Thermo-gravimetric analysis of tape specimens and combustion tests in model and full-scale roadways at the Kyushu Coal and Mine Technology Research Centre in Japan revealed a qualitative relationship between the combustion characteristics of tape in a shaft and its thermogravimetric characteristics^3^. Wang Shingshen et al. summarized the tendency of spontaneous coal combustion and analyzed the ignition properties and characteristics of several common solid combustible materials in underground settings during fire incidents, He also investigated conditions leading to smoke flow rollback in level-channel fires, derived a conditional formula for its occurrence, and introduced two meaningful dimensionless criteria^4^. Oka et al. investigated the smoke transport behavior in roadway fires using a horizontal roadway experimental model and derived a formula to calculate the critical wind speed^5^. Wang Deming et al. discovered the obstacle effect of burning flames on wind flow in roadways and its impact on throttling, On the basis of this, he proposed the concept of fire zone resistance, which encompasses resistance generated by thermal expansion of smoke flow and local resistance caused by wind flow obstruction by flame in the fire zone^7^. Alongside Zhou Fubao et al., we obtained the fitting equations for dimensionless counterflow length, considering the heat release rate of the fire source and roadway wind speed through experiments^8^. Subsequent research employed and reproduced the smoke flow rolling back phenomenon of the roadway fires through CFD technology to reproduce smoke flow rollback phenomena in roadway fires and predict rollback distances under varying conditions of heat release rate and ventilation speed^14^. By simulating fire situations in roadways with different inclinations and varying ventilation wind speeds, Jian Liu determined the laws governing smoke flow countercurrent length under the influence of wind speed and roadway inclination^16^. Zhu Hongqing et al. investigated backflow length and critical velocity of smoke in main roadways when a branching roadway fire occurred in different fire locations. It was concluded that the branch roadway fires have lower backflow length and critical velocities compared with single-hole roadway fires. The above scholars have extensively researched and summarized the combustion characteristics of ignition sources in mine fires and their effects on mine ventilation^24^.

When an actual fire occurs in a mine, the traditional numerical simulation method for predicting the fire’s evolution within the mine involves modeling, environmental parameter setting, arithmetic, and data processing. The entire process takes over 24 h, making it impractical to address emergencies and ensure timely disaster response and evacuation. Consequently, traditional numerical modeling methods are ill-suited to meet the urgent requirements of such situations.

In recent years, as computer processing speed and storage capacity have advanced, neural networks with multiple hidden layers have demonstrated their capability to develop swift prediction models for roadway fire scenarios. Initially, neural networks were prone to overfitting and slow parameter training, particularly when handling substantial volumes of data. However, with the advancement of computer processing speed and storage capacity, Professor Hinton introduced two important points: firstly, Neural networks with multiple hidden layers can effectively capture inherent data features, that can portray the intrinsic attributes of the data, which is helpful for aiding tasks like data visualization and classification; and secondly, the difficulty in training deep neural networks can be overcome with the help of an unsupervised "layer-by-layer initialisation" strategy. At the same time, a two-stage strategy based on "layer-by-layer pre-training" and "fine-tuning" is proposed to solve the problem of network parameter training in deep learning. Improved AI^25–28^ is capable of fast prediction after training. Liu Jian et al. applied neural networks to mine research^29^ to establish a machine learning model for swiftly predicting the propagation of gas explosion causative factors and achieved better prediction results. Other researchers^30–33^ have also achieved better results by integrating neural networks into various aspects of mining research.

This paper aims to improve mine disaster emergency response decision-making by integrating FDS simulation and BP neural network modeling. We first conducted FDS simulations in a wide range of roadway fires under different conditions. Subsequently, a BP neural network is trained using a subset of the FDS simulation results as training samples, while the remaining results are compared with predicted values to verify the neural network’s accuracy. The trained BP neural network demonstrates the capability to predict roadway fires under varying conditions rapidly. This predictive capacity can be seamlessly integrated into the mine's intelligent ventilation system, enabling swift emergency response decisions based on the prediction results. Such measures serve to safeguard the safety of people's lives and property.

## Mine fire modeling

### Principles of FDS simulation

The combustion simulation software employed in this study is FDS, featuring a computational model that offers an approximate of the N-S equations. It excels in handling low Mach number flows. Furthermore, the N–S equations are processed using a spatial filtering method, which filters out minor pressure variations at high frequencies while preserving significant alterations in physical attributes like pressure, temperature, and flow rate. FDS obtains its results by solving three conservation equations for mass, momentum, and energy, in addition to the component equations. The four equations are as follows:

Mass conservation equation:

1 $$\frac{\partial \rho }{{\partial t}} + \nabla \rho v = 0$$

Momentum conservation equation:

2 $$\rho \left( {\frac{\partial v}{{\partial t}} + \left( {v\nabla } \right)v} \right) + \nabla p = \rho g + f + \nabla \tau$$

Energy conservation equation:

3 $$\frac{\partial }{{\partial {\text{t}}}}\left( {\rho h} \right) + \nabla \rho hv = \frac{Dp}{{Dt}} - \nabla k\nabla T + \sum {\nabla h_{l} } \rho D_{l} \nabla Y_{l}$$

Component equations:

4 $$\frac{\partial }{{\partial {\text{t}}}}\left( {\rho Y_{l} } \right) + \nabla \left( {Y_{l} v} \right) = \nabla \left( {\rho D_{l} \nabla Y_{l} } \right) + m_{l}^{\prime \prime \prime }$$

where $\frac{Dp}{{Dt}}$ is the rate of change of pressure over time, as expressed by the following formula:

5 $$\frac{Dp}{{Dt}} = \frac{\partial p}{{\partial t}} + v\nabla p$$

By using the thermodynamic equation of state to supplement the set of conservation equations, the pressure of an ideal gas can be approximated by decomposing it into the following three components: static pressure, dynamic pressure, and potential pressure:

6 $$p = p_{0} + \rho gz + p_{v}$$

7 $$p_{0} = \rho TR\sum {\left( {{{Y_{l} } \mathord{\left/ {\vphantom {{Y_{l} } {M_{l} }}} \right. \kern-0pt} {M_{l} }}} \right)} = \rho {{TR} \mathord{\left/ {\vphantom {{TR} M}} \right. \kern-0pt} M}$$

where $\rho$ is the density of air,${{kg} \mathord{\left/ {\vphantom {{kg} {m^{3} }}} \right. \kern-0pt} {m^{3} }}$; $t$ is time,s; $v$ is the wind speed,m/s; $p$ is the gas pressure,Pa; $g$ is gravitational acceleration,$9.81{m \mathord{\left/ {\vphantom {m {s^{2} }}} \right. \kern-0pt} {s^{2} }}$; for $f$ source items arising from heat and pollution sources, N/m^3^; $\tau$ is stress tensor,${\text{N}}$; $h$ is enthalpy,${J \mathord{\left/ {\vphantom {J {kg}}} \right. \kern-0pt} {kg}}$; $k$ is the thermal conductivity,${{\text{w}} \mathord{\left/ {\vphantom {{\text{w}} {\left( {{\text{m}} \cdot {\text{K}}} \right)}}} \right. \kern-0pt} {\left( {{\text{m}} \cdot {\text{K}}} \right)}}$; $T$ is temperature, K; $D_{l}$ is the diffusion coefficient,${{m^{2} } \mathord{\left/ {\vphantom {{m^{2} } s}} \right. \kern-0pt} s}$; $Y_{l}$ is the mass fraction of the first component; $m_{l}^{\prime \prime \prime }$ is the rate of production of the first mass in a single volume, kg/m^3^; $R$ is the gas molar constant,${{\text{J}} \mathord{\left/ {\vphantom {{\text{J}} {{\text{mol}} \cdot {\text{k}}}}} \right. \kern-0pt} {{\text{mol}} \cdot {\text{k}}}}$; $M_{l}$ is the molar mass of the lth component,${{kg} \mathord{\left/ {\vphantom {{kg} {mol}}} \right. \kern-0pt} {mol}}$.

### Geometric modeling

We created a rectangular roadway model with a length of 2000 m, a width of 5 m, and a height of 4.5 m. The ignition source is positioned at 800 m from the entrance of the roadway. The roadway wall temperature is set at 30 °C, and the relative humidity is 40 percent. The walls are constructed from concrete with a modeled wall thickness of 0.5 m. Concrete has a thermal conductivity of 1.8w/(m*K) and a specific heat capacity of 1.04 kJ/(kg*K). The roadway entrances are set up as supply surfaces to provide uniform airflow with adjustable temperature and velocity. The exit of the roadway is designated as an open surface with free outflow and dynamic pressure of 0 Pa. The ignition process starts after a 10-s pre-ventilation period, and the total simulation duration is 650 s. The fire is simulated at the exit of the roadway. Length of each simulation is 36 h.

### Fire source setting

The n-heptane combustion reaction was used as a replacement for the diesel combustion reaction (The combustion reaction mechanism of n-heptane is relatively mature and its 16-alkane number is similar to that of diesel), and the combustion model utilized is as follows:

8 $$Q = \phi \cdot m \cdot \Delta H$$

where *Q* is heat release rate, $\phi$ is a combustion efficiency factor reflecting the degree of incomplete combustion in a fire, dimensionless;$m$ is the rate of combustion of the mass of the combustible,${{kg} \mathord{\left/ {\vphantom {{kg} {(m^{2} }}} \right. \kern-0pt} {(m^{2} }} \cdot s)$; $H$ is the calorific value of the combustible material,${{kJ} \mathord{\left/ {\vphantom {{kJ} {kg}}} \right. \kern-0pt} {kg}}$.

**Figure 1:**
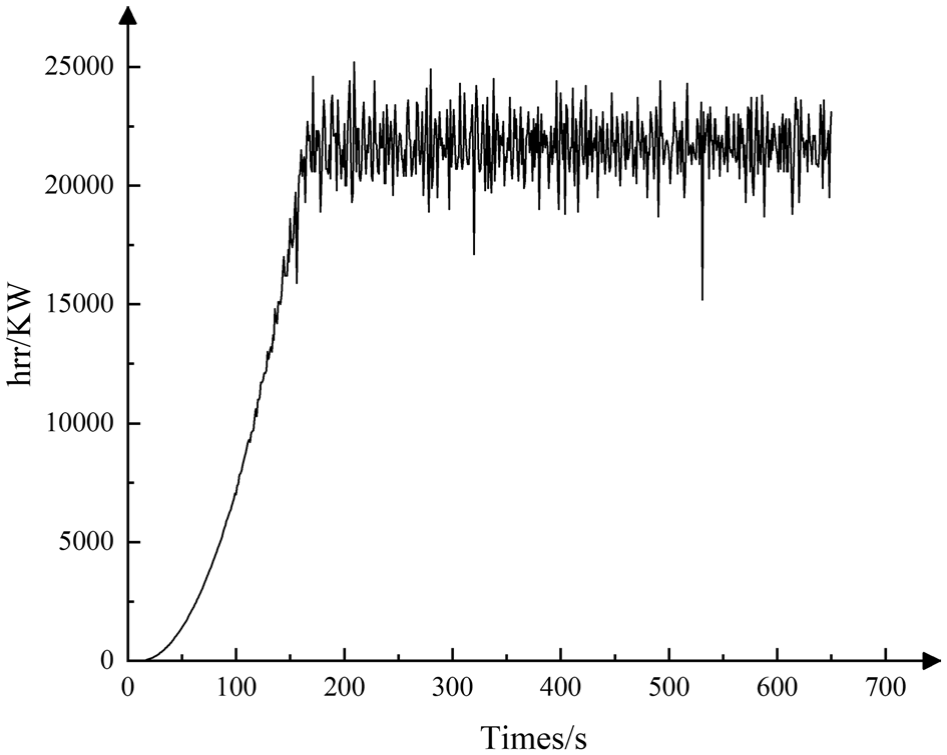
Fire source heat release modeling diagram.

Assume the combustion efficiency factor $\phi$ as 1. The *t*^2^ model was chosen for the combustion model of the fire source, and the variation of the heat release rate is shown in Fig. 1.

### Mesh size selection

The FDS Guidebook uses ${{D^{*} } \mathord{\left/ {\vphantom {{D^{*} } \delta }} \right. \kern-0pt} \delta }$($D^{*}$ is the fire feature diameter and $\delta$ is the grid size) to assess the reasonableness of the grid size.$D^{*}$ is defined as:

9 $$D^{*} = \left( {\frac{Q}{{\rho c_{p} T\sqrt g }}} \right)^{\frac{2}{5}}$$

where $Q$ is the rate of heat release from the ignition source in the unit of KW; $c_{p}$ is the specific heat of air at constant pressure,${{kJ} \mathord{\left/ {\vphantom {{kJ} {\left( {kg \cdot K} \right)}}} \right. \kern-0pt} {\left( {kg \cdot K} \right)}}$; $T$ is the initial ambient temperature, K.

As per the manual, the reliability of the simulation results is considered high when the value of ${{D^{*} } \mathord{\left/ {\vphantom {{D^{*} } \delta }} \right. \kern-0pt} \delta }$ is between 4 and 16, and the mesh size is within the range of 0.0625 $D^{*}$ to 0.25 $D^{*}$. The heat release rate of the fire source for this simulation ranges from 14.45 to 28.90, and the characteristic diameter of the fire source ranges from 2.96 m to 3.9 m. Consequently, to maintain the reliability of the simulation, the grid size should be set between 0.185 m and 0.975 m.

**Figure 2:**
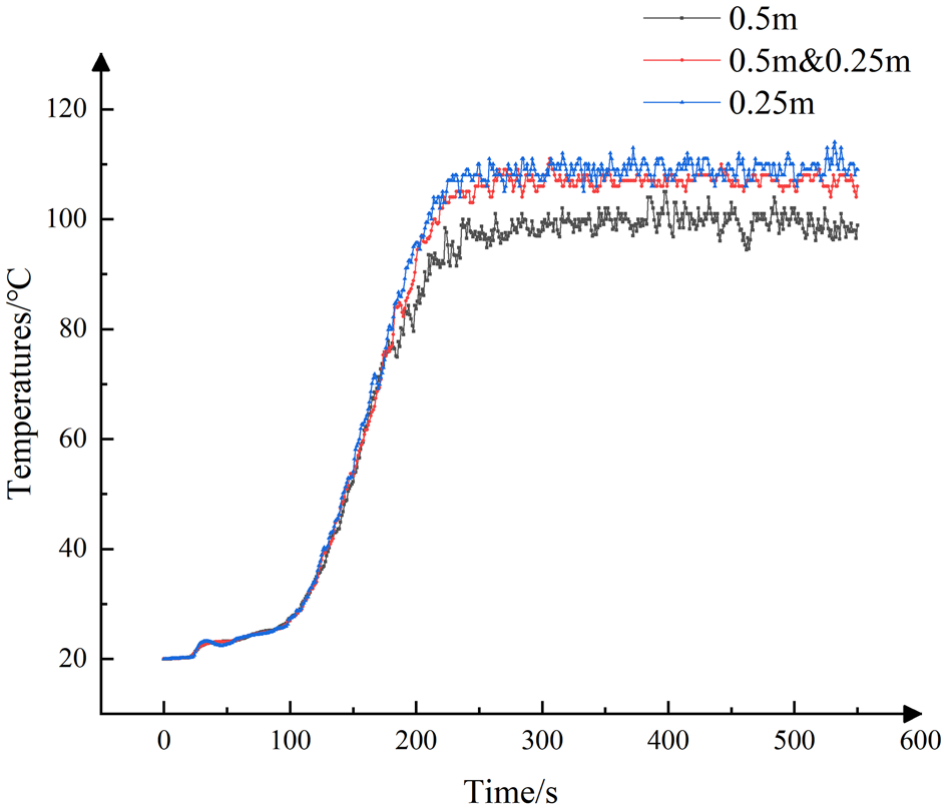
Simulation results for different mesh sizes.

In this simulation, the grid size was initially set to 0.5 m, and local encryption was carried out within 100 m before and after the fire source thus the encrypted grid size was 0.25 m. This resulted in a total of total 306,000 grids. To ensure that the chosen grid size is suitable, simulations were conducted using different grid sizes for the same environmental roadway. Grid sizes of 0.5 m and 0.25 m were selected for comparative verification. Temperature data from measurement points located 200 m below the fire source and at a height of 2.25 m were used for this comparison, as shown in Fig. 2. The results show that the selected grid size generally meets the accuracy requirements of the simulation and is indeed feasible.

### Experimental design of lane fire simulation

The experiment numbers, along with the corresponding environmental parameters for the lanes, are shown in Table 1.

**Table 1:** Parameter for each experiment.

Number	Fire intensity (L/s)	Roadway section area (m^2^)	Roadway inlet air velocity (m/s)	Roadway inclination (°)	Ventilation temperature (°C)	Atmospheric pressure (Pa)
1	0.5	22.5	2.18	0	20	101,325
2	0.75	22.5	2.18	0	20	101,325
3	1	22.5	2.18	0	20	101,325
4	0.75	18	2.18	0	20	101,325
5	0.75	22.5	3.09	0	20	101,325
6	0.75	22.5	6.75	0	20	101,325
7	0.75	22.5	2.18	2	20	101,325
8	0.75	22.5	2.18	6	20	101,325
9	0.75	22.5	2.18	10	20	101,325
10	0.75	22.5	2.18	14	20	101,325
11	0.75	22.5	2.18	− 2	20	101,325
12	0.75	22.5	2.18	− 6	20	101,325
13	0.75	22.5	2.18	− 10	20	101,325
14	0.75	22.5	2.18	− 14	20	101,325
15	0.75	22.5	2.18	0	2	101,325
16	0.75	22.5	2.18	0	10	101,325
17	0.75	22.5	2.18	0	20	115,000
18	0.75	22.5	2.18	0	20	80,000
19	0	22.5	2.18	0	20	101,325
20	0.6	22.5	2.18	0	20	101,325
21	0.7	22.5	2.18	0	20	101,325
22	0.8	22.5	2.18	0	20	101,325
23	0.75	22.5	9	0	20	101,325
24	0.75	22.5	10	0	20	101,325
25	0.75	22.5	2.18	1	20	101,325
26	0.75	22.5	2.18	3	20	101,325
27	0.75	22.5	2.18	5	20	101,325
28	0.75	22.5	2.18	7	20	101,325
29	0.75	22.5	2.18	11	20	101,325
30	0.75	22.5	2.18	12	20	101,325
31	0.75	22.5	2.18	13	20	101,325
32	0.75	22.5	2.18	16	20	101,325
33	0.75	22.5	2.18	18	20	101,325
34	0.75	22.5	2.18	20	20	101,325
35	0.75	22.5	2.18	− 7	20	101,325
36	0.75	22.5	2.18	− 8	20	101,325
37	0.75	22.5	2.18	− 9	20	101,325
38	0.75	22.5	2.18	− 11	20	101,325
39	0.75	22.5	2.18	− 12	20	101,325
40	1	18	3	5	20	101,325
41	0.5	22.5	12	5	20	101,325
42	0.75	22.5	10	8	20	101,325
43	0.5	22.5	4	3	20	101,325
44	1	22.5	6	− 6	20	101,325

### Raw data acquisition and processing

**Figure 3:**
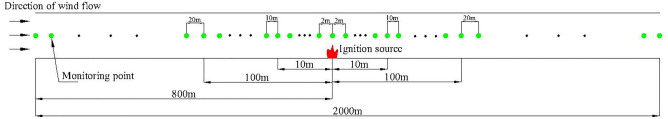
Schematic diagram of roadway monitoring arrangement points.

The monitoring points are strategically placed to capture various parameters at different times and locations in the roadway, and the data is presented in tabular form. These monitoring points include temperature, visibility, CO concentration, density, and other variables, all positioned at the center of the roadway height of 2.25 m. The arrangement of monitoring points is (1) every 20 m from the entrance of the airflow into the roadway, (2) every 10 m before and after 100 m of the fire source, and (3) every 2 m before and after 10 m of the fire source. The monitoring points record data at one-second intervals. The layout of these monitoring points is visually depicted in Fig. 3 for reference.

Temperature, visibility, CO concentration, density, and other data were recorded from the designated monitoring points. Given the substantial volume of data, the training data for the neural network was down sampled at 30-s intervals. Additionally, the fire wind pressure was calculated using the Eq. (10), which is widely used in many applications:

10 $$h_{f} = gZ\left( {\rho_{0} - \rho_{s} } \right)$$

where $h_{f}$ is the alley fire wind pressure, Pa; $Z$ is the height difference between the entrance and exit ends of the roadway, m; $\rho_{0}$ and $\rho_{s}$ are the global densities of airflow in front of the roadway before and after the fire, respectively,${{kg} \mathord{\left/ {\vphantom {{kg} {m^{3} }}} \right. \kern-0pt} {m^{3} }}$.

### Demonstration of the simulation results of alleyway fires

**Figure 4:**
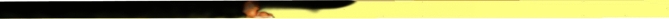
Graph of simulation results of alleyway fires.

In the event of a fire within a roadway, the release of high-temperature smoke and toxic gases leads to their dispersion to the downwind side of the fire source, carried by the wind flow. This phenomenon significantly reduces visibility on the downwind side of the fire source and presents a significant obstacle to the safe evacuation of personnel. Moreover, with the continuous generation of smoke, it starts to flow backward, spreading to the upper section of the roadway on the upwind side of the fire source. This dynamic is depicted in Fig. 4.

## Neural network model and parameter settings

**Figure 5:**
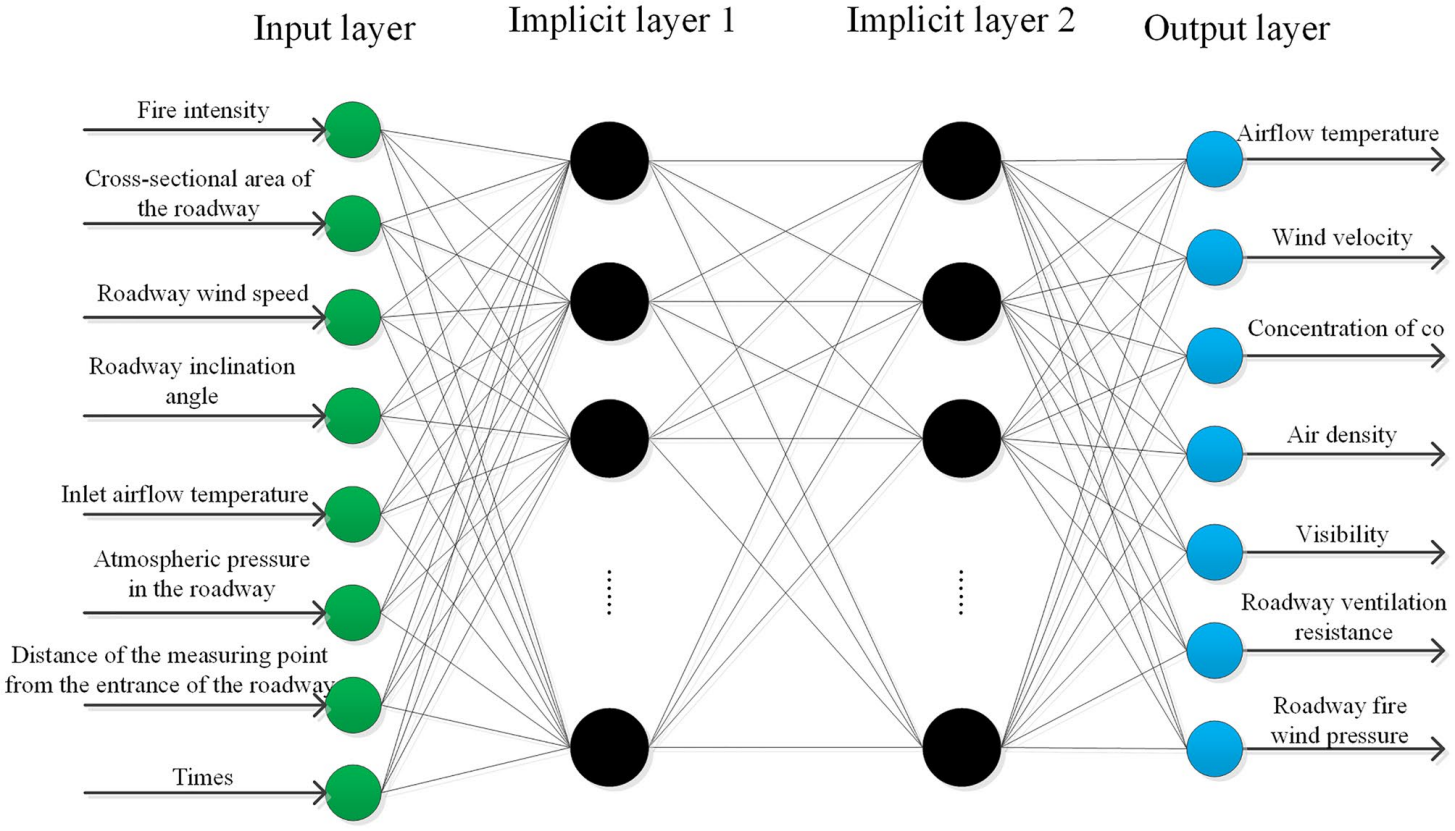
Neural network training model diagram.

The parameters, including fire intensity, inlet wind speed (Inlet wind speed when the roadway is not on fire), roadway inclination angle (with positive values indicating upward ventilation and negative values indicating downward ventilation), time, etc. are used as the input layer of the neural network. The required parameters such as air temperature, CO concentration, and fire wind pressure in the roadway are output, as shown in Fig. 5.

### Positive transfer of network input information (input layer to output layer)

Raw data normalization:

11 $$x = \frac{{x - x_{\min } }}{{x_{\max } - x_{\min } }}$$

The output of the *i*th node of the input layer is:

12 $$x_{i} ,(i = 1,2,...,8)$$

The output of the *j*th node of the implicit layer 1 is:

13 $$y_{j} = f_{1} \left( {\sum {_{i = 1}^{8} } w_{ij} \cdot x_{i} + b_{j} } \right),\left( {j = 1,2,...22} \right)$$

The output of the *k*th node of the implicit layer 2 is:

14 $$y_{k} = f_{2} \left( {\sum {_{j = 1}^{22} } w_{jk} \cdot y_{j} + b_{k} } \right),\left( {k = 1,2,...22} \right)$$

The output of the *m*th node of the output layer is:

15 $$y_{m} = f_{3} \left( {\sum {_{k = 1}^{22} } w_{jm} \cdot y_{k} + b_{m} } \right),\left( {m = 1,2,...7} \right)$$

where $w_{ij}$ is the connection weight between the *i*th node of the input layer and the *j*th node of the implicit layer 1; $b_{j}$ is the threshold on the *j*th node of implicit layer 1; $f_{1}$ is the excitation function for implicit layer 1; $y_{j}$ is the output of the *j*th node of implicit layer 1; $w_{jk}$ is the connection weight between the *j*th node of implicit layer 1 and the* k*th node of implicit layer 2; $b_{k}$ is the threshold on the *k*th node of implicit layer 2; $f_{2}$ is the excitation function for implicit layer 2; $y_{k}$ is the output of the *k*th node of implicit layer 2; $w_{km}$ is the connection weight between the *k*th node of the implicit layer 2 and the *m*th node of the output layer; $b_{m}$ is the threshold on the *m*th node of the output layer; $f_{3}$ is the excitation function of the output layer; $y_{m}$ is the output of the *m*th node of the output layer.

### Backpropagation of network computational error (output layer to input layer)

Its mathematical expression is:

16 $$E\left( {w,b} \right) = {1 \mathord{\left/ {\vphantom {1 N}} \right. \kern-0pt} N} \cdot \sum {\left( {o_{m} - y_{m} } \right)}^{2}$$

where $o_{m}$ is the expected results of the *m*th node of the output layer, and $N$ is the number of samples.

The computational errors of the network are initially transmitted from the output layer to hidden layer 2, where the weights and thresholds between the output layer and the hidden layer 2 are adjusted. The correction is carried out in the direction of the negative (inverse) gradient between variables $E$ and $w$. The expression for the correction quantity $\Delta w_{km} \left( n \right)$ is:

17 $$\Delta w_{km} \left( n \right) = - \eta \frac{\partial E\left( n \right)}{{\partial w_{km} \left( n \right)}}$$

where $\eta$ is the learning efficiency of the network, and $n$ is the number of iterations.

The adjusted weights $w_{km} \left( {n + 1} \right)$ can be expressed as follows:

18 $$w_{km} \left( {n + 1} \right) = w_{km} \left( n \right) + \Delta w_{km} \left( n \right)$$

Similarly, the correction $b_{m} \left( {n + 1} \right)$ for threshold $b_{m} \left( n \right)$ can be expressed as:

19 $$b_{m} \left( {n + 1} \right) = b_{m} \left( n \right) + \Delta b_{m} \left( n \right)$$

The computational error is propagated from the output layer through hidden layer 2 and is subsequently passed to the input layer through hidden layer 1. Weights and thresholds are adjusted accordingly during the process. Once the specified number of iterations has been completed, the computation is terminated, and the final correction weights and thresholds are saved with the model for subsequent recall during testing.

## Training and result analysis of neural network models

### Neural network model training

**Figure 6:**
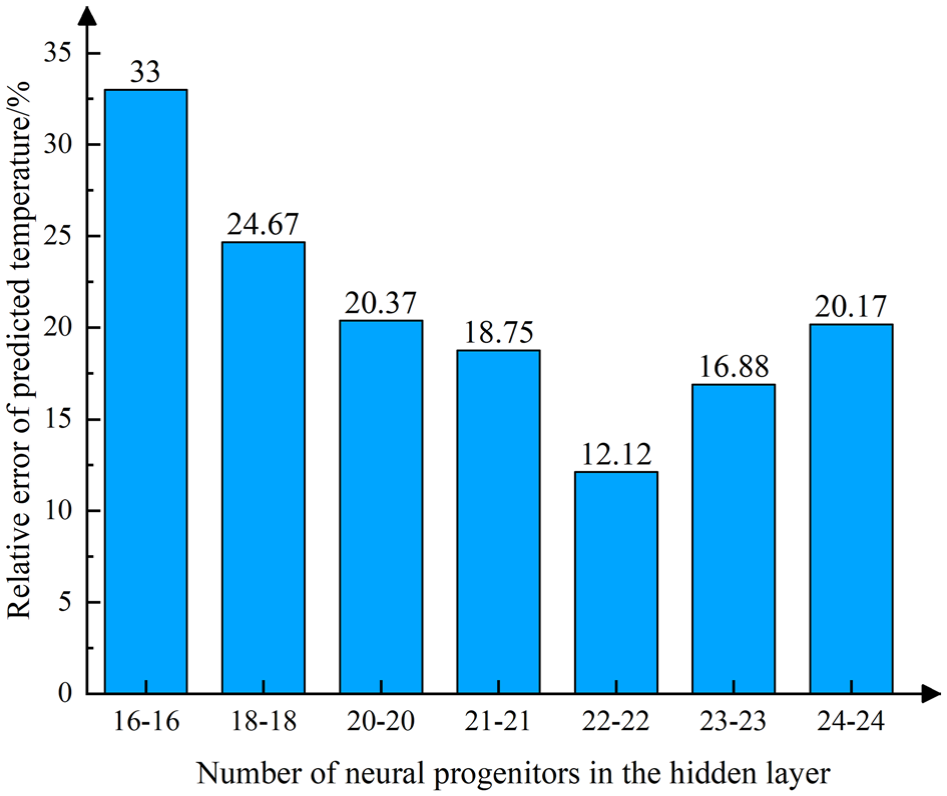
Error plots of models with different numbers of hidden layers.

**Figure 7:**
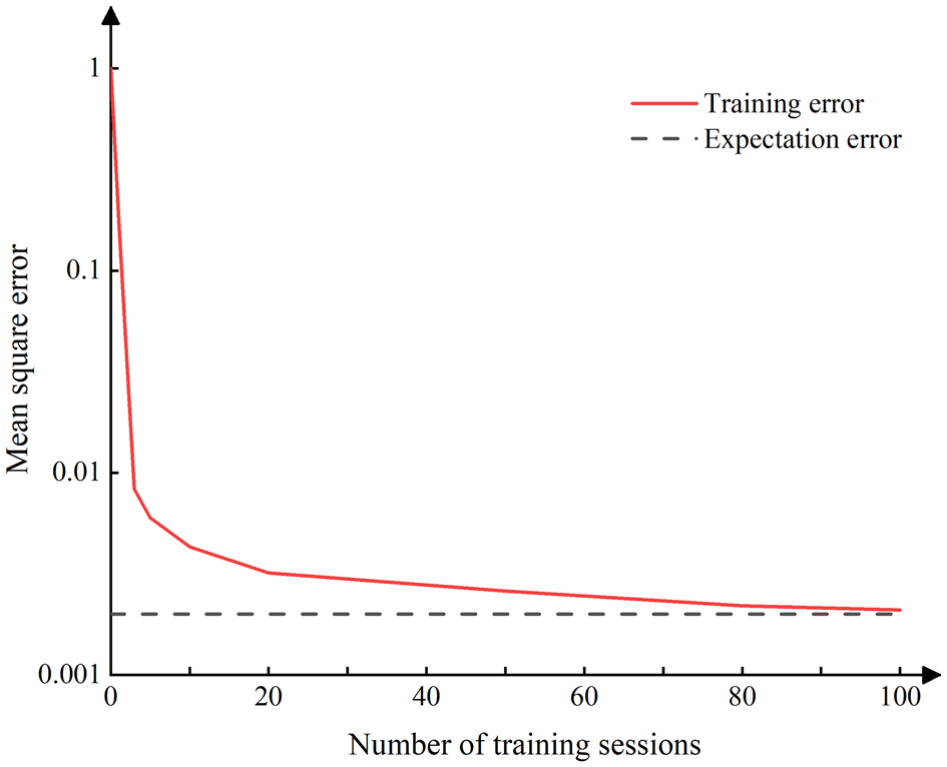
Convergence plot of the model training process.

The results of the FDS simulation were transformed into the format required by the neural network. Out of the available data, 36 sets were randomly chosen for training, while 8 sets were reserved for testing. Through iterative testing and adjustment, the ReLU (rectified linear unit) function was ultimately selected as the activation function for the implicit layers, and the liner function was chosen as the activation function of the output layer. The relative error in predicting temperatures for models with different numbers of neural progenitors in the hidden layers is shown in Fig. 6. For minimum errors, the number of neural progenitors per layer in the hidden layers is set to 22. The convergence of the model is achieved after 100 iterations, The length of each training session is 20 min. as shown in Fig. 7.

### Training results and analysis

The mathematical error analysis of the predicted results with the simulated results is shown in Table 2.

**Table 2:** Error table for prediction results.

	Average relative error /%	Average absolute error
Temperature	12.12	4.91 °C
Concentration of CO	–	3.49 ppm
Air density	2.9	0.0 3kg/m^3^
Visibility	10.44	0.87 m
Fire and wind pressure	10.39	16.78 Pa

**Figure 8:**
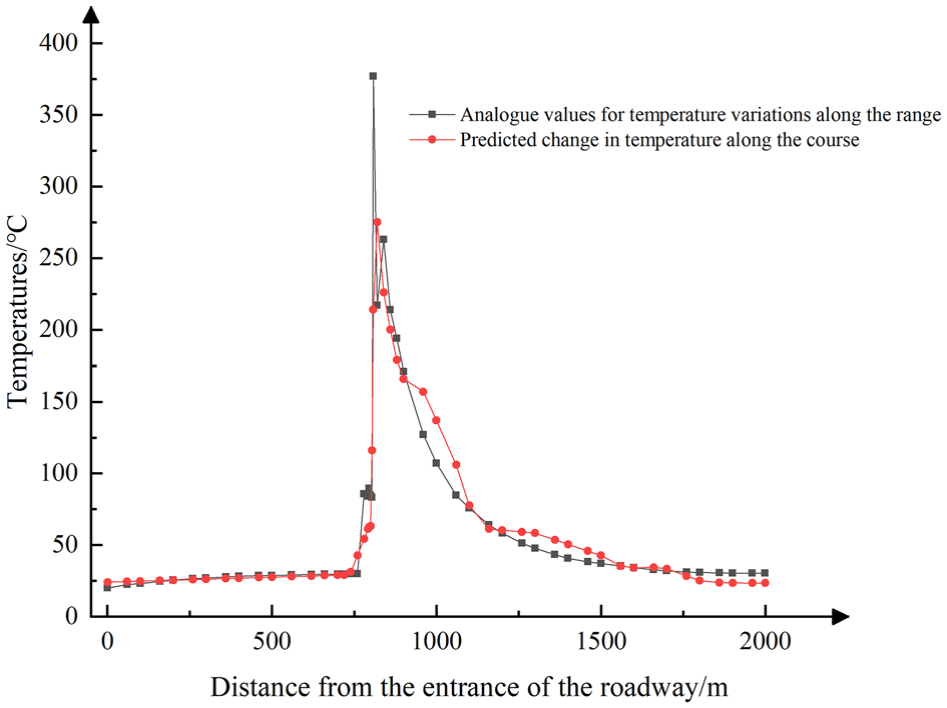
Temperature profile with distance in mine fire roadway.

**Figure 9:**
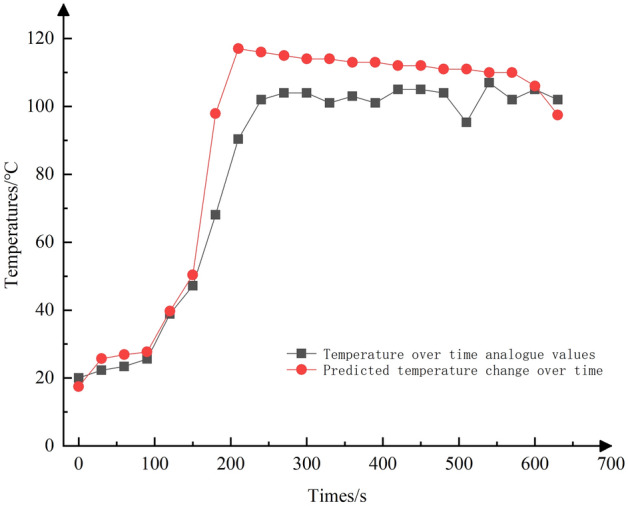
Comparison of temperature variation with time.

**Figure 10:**
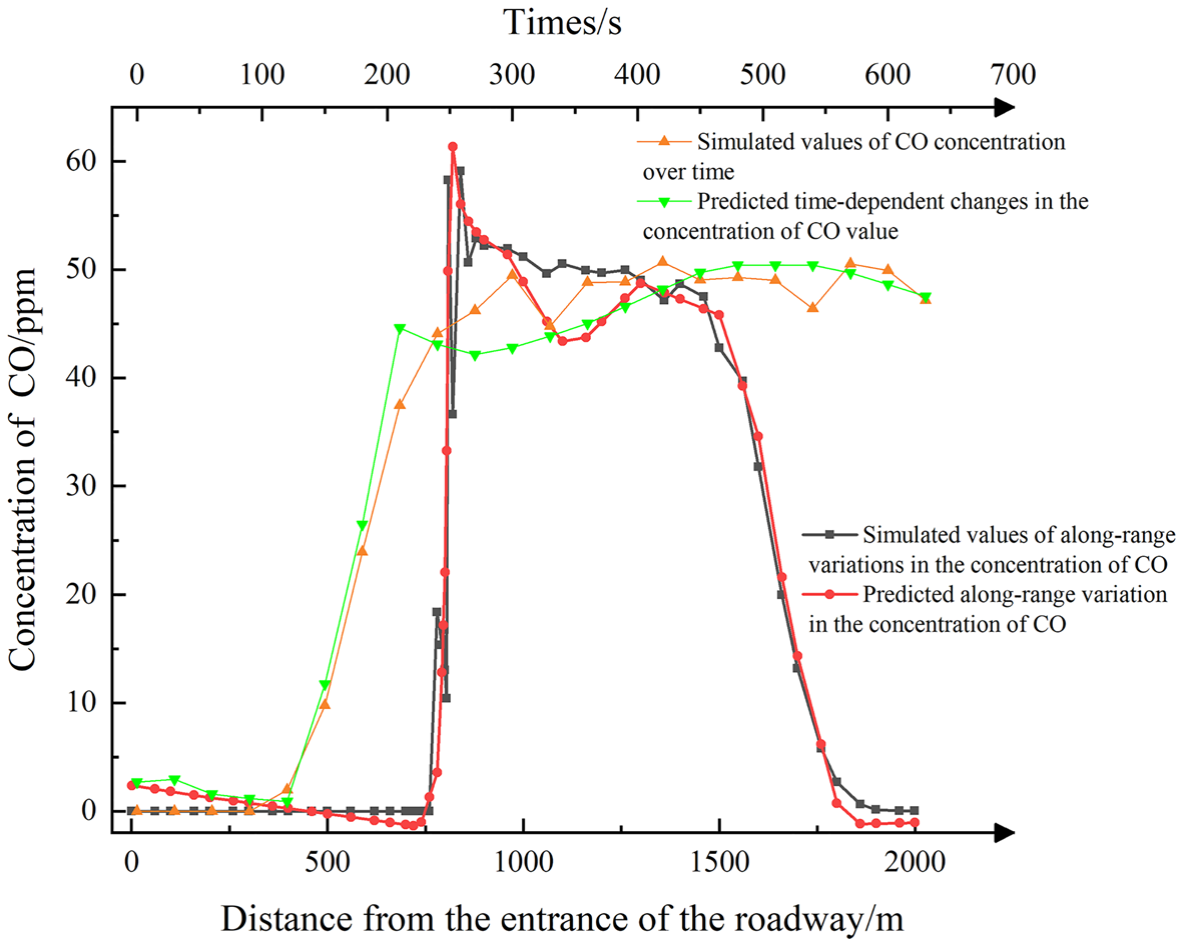
CO concentration comparison plot.

**Figure 11:**
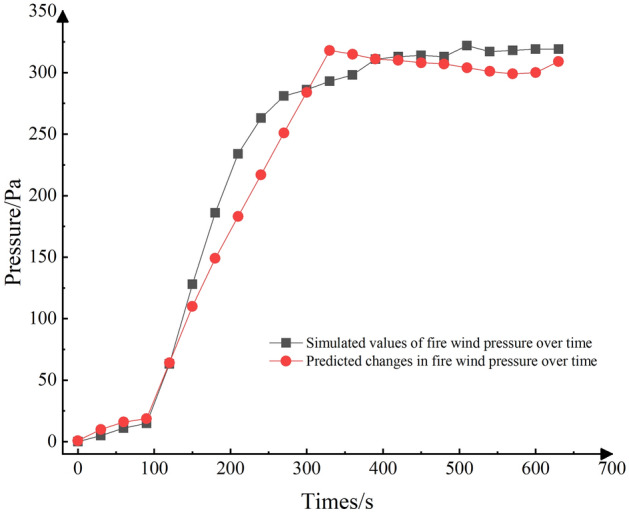
Fire and Wind Pressure Comparison.

**Figure 12:**
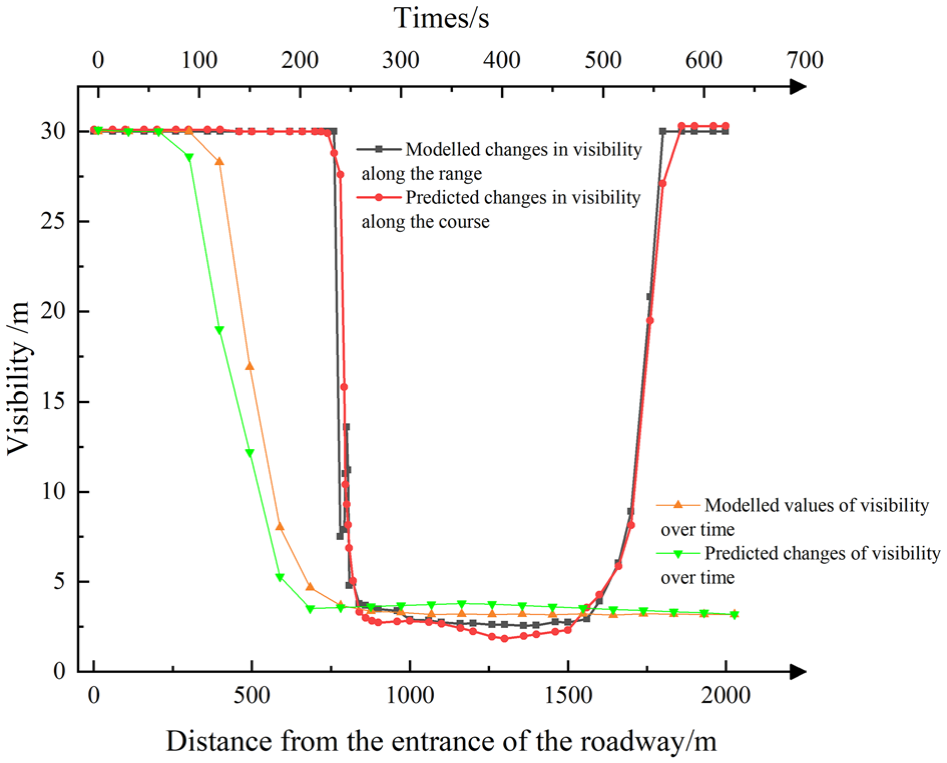
Visibility comparison map.

**Figure 13:**
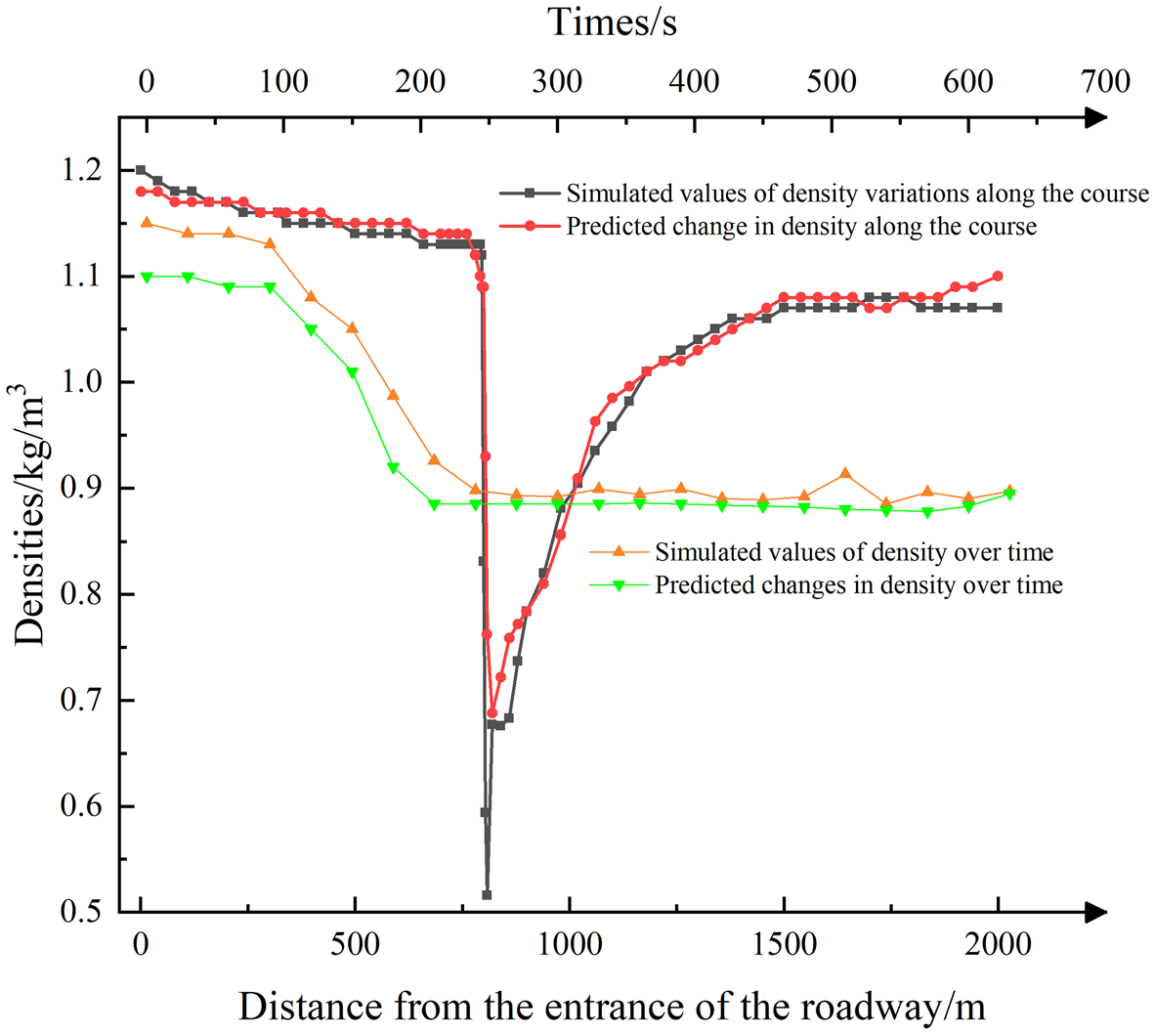
Density comparison chart.

The prediction results of a randomly selected set of training data at different moments on the downwind side of the fire source at 200 m (1000 m from the entrance of the wind flow) and at different distances at 450 s are shown in Figs. 8, 9, 10, 11, 12, 13.

As shown in Fig. 8, the temperature rises sharply at a distance of 20 m (equivalent to 780 m from the roadway entrance) on the upwind side of the fire source. It reaches its peak at 8 m (approximately 808 m from the entrance) on the downwind side of the fire source, and begins to decline, stabilizing at around 30 °C. The prediction accuracy is notably higher within the 0–780 m interval, with an average error of approximately 3%. However, in the 780–1500 m interval, the prediction error increases to 19.8%. This variation can be attributed to the significant alterations in ambient temperature caused by the intense heat radiation from the fire source and the presence of high-temperature smoke, making predictions more challenging in this region. Figure 9 demonstrated that the prediction error is more pronounced during the period when the fire source is evolving from its early stages to full development. However, as the fire reaches its fully developed stage, the predictions become increasingly accurate.

As illustrated in Fig. 10, the average absolute error in the prediction of CO concentration with respect to distance is 1 ppm for the intervals of 0–780 m and 1300–2000 m, and 6.58 ppm for the interval of 780–1300 m. When it comes to predicting CO concentration over time, the predicted value fluctuates. Nevertheless, the overall prediction results are relatively accurate.

As depicted in Fig. 11, the prediction accuracy in the early growth stage of the fire source is relatively low, primarily due to the fire source being in a dynamic expansion stage. During this phase, the air within the roadway undergoes rapid heating, causing it to expand. Consequently, the gas density in the roadway decreases rapidly, resulting in a rapid increase in fire wind pressure. The magnitude of these changes in this interval is substantial, making it challenging to predict accurately.

Observing Fig. 12, changes in visibility are primarily influenced by the behavior of smoke flow. The retrogression of smoke flow to 780 m leads to a significant reduction in visibility, while at 1760 m, the smoke is diluted, resulting in an increase in visibility.

The prediction results of visibility change with distance (Fig. 12), closely align with the fitting results. However, the average relative error remains at 10.44%. This discrepancy can be attributed to the fact that in areas where the smoke has spread, the visibility is only about 3 m. In such low-visibility conditions, even a minor difference between the predicted and simulated values can lead to an amplified relative error, thus increasing the overall average relative error.

Figure 13 illustrates that as the air heats up, it causes the gas to expand, leading to a decrease in density. The reduction in density is more pronounced in the area directly above the fire source due to the direct heating of the air by the fire, resulting in rapid expansion. As one moves further away from the fire source, the density increases gradually. However, it's important to note that the density is lower on the downwind side of the fire source compared to the upwind side, primarily due to the heating effect of the high-temperature smoke plume. In terms of predictions with respect to distance, they generally align well with the fitted data. However, time-dependent predictions tend to be less accurate during the early growth phase of the fire source. This is because the fire source takes longer to fully develop during this phase, and the lower density at this stage has a more significant impact on the model training, resulting in overall lower predictive accuracy.

**Figure 14:**
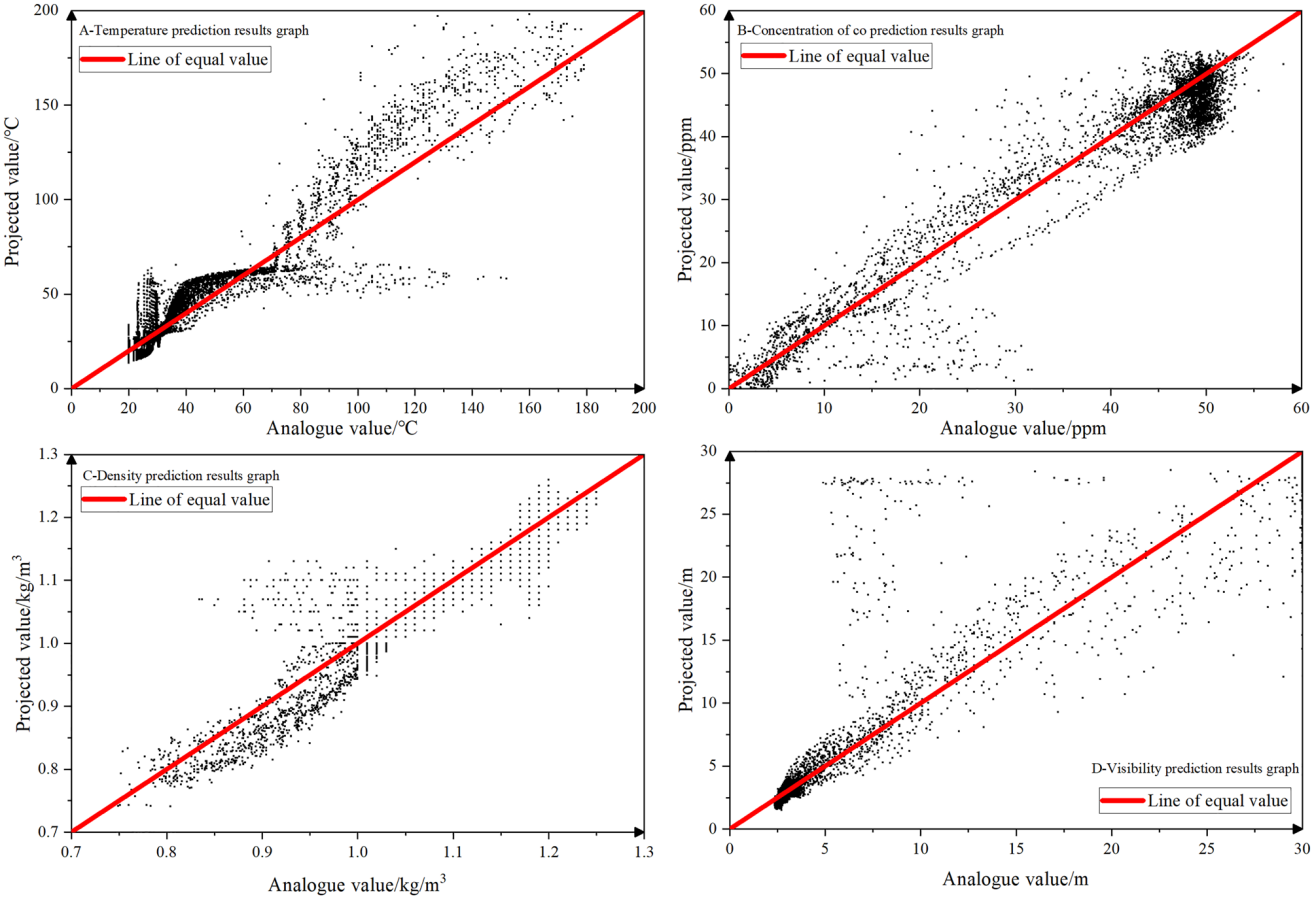
Chart of predicted results.

**Figure 15:**
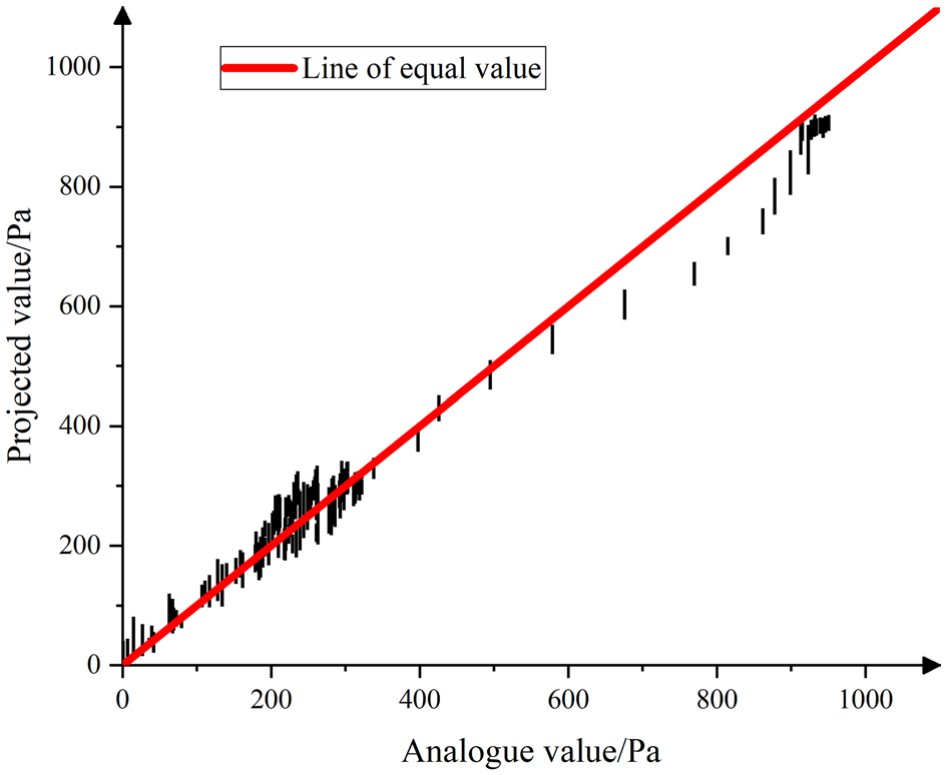
Fire wind pressure prediction results.

Considering that the environmental conditions in the vicinity of the fire source attachment hold limited research significance for emergency escape, the simulation results are utilized as the horizontal coordinate, while the prediction results are used as the vertical coordinate. This approach is employed to generate Figs. 14 and 15, encompassing all the data except for the region extending from 10 m upwind of the fire source to 100 m downwind of the fire source.

Figure 14A reveals that most of the roadway temperatures are below 50 °C, and the corresponding predicted values are all also below 50 °C. While the predicted temperatures tend to be higher than the simulated values, they remain within the contour boundaries. This discrepancy arises from the exceptionally high temperature at the fire source attachment, which leads the neural network model to assign a larger weight to this data to fit it better, thus influencing the overall squared absolute error.

In Fig. 14B, it is apparent that the CO concentration exhibits a larger prediction error in the region with higher simulated values (downwind side of the fire source). Nevertheless, the predictions remain generally within the contour boundaries. Since CO concentration on the upwind side of the roadway is essentially zero, a large number of data points converge around the origin attachment.

Figure 14C shows that in areas where the smoke flow spreads, visibility is reduced to approximately 4 m. The predicted results closely match this value, except for a slight deviation when the visibility is 30 m in the area where the smoke flow is not spreading. Overall, the predicted results align well with the contour boundaries, indicating greater accuracy.

Figure 14D shows a matrix-like simulation in the region of simulated values ranging from 1.0 to 1.3 kg/m^3^, indicating a certain degree of overfitting. However, the predictions still fall within the contour annexes and are considered usable.

In general, the prediction results closely match the simulation values, particularly when the fire wind pressure is low (< 600 Pa). However, when the wind pressure falls within the range of 600 to 1000 Pa, the prediction results tend to be lower than the simulated values. This deviation can be attributed to the data source, which is derived from the simulation results of a roadway with a 20° inclination angle—an extreme case. Consequently, the neural network assigns lower weights to this data, resulting in less accurate prediction results. Nevertheless, it's worth noting that the majority of the prediction results align closely with the isobar, indicating the overall quality of the predictions.

This paper focuses solely on the fire in a single roadway, and only the midpoint data of the roadway section height is selected to align with emergency relief needs. Subsequent research could explore other heights for the study of smoke flow reversal and roadway wind flow reversal. The key achievement of this study is the ability to instantaneously predict the environmental conditions of the roadway at any given time and location when a fire occurs under different conditions. This breakthrough holds significant importance for both mine fire research and emergency relief efforts.

## Conclusion

In this study, a wide range of roadway fire combustion scenarios were simulated using FDS simulation software, covering a comprehensive set of environmental scenarios of mine roadways. The neural network model was trained on this data, leading to the following outcomes:

1. The trained neural network model can quickly predict environmental parameters at any time and location within the roadway.
2. The average relative error in the prediction results was 12.12% for temperature, with an average absolute error of 0.87 m for visibility, 3.49 ppm for CO concentration, 16.78 Pa for fire wind pressure, and 2.9% for density.
3. Predictions are less accurate in the vicinity of the fire source due to the higher temperatures and increased turbulence in the air flow, resulting in significant parameter variations. Therefore, predictions are more reliable farther away from the fire source than in the fire annex.

## Data Availability

The datasets generated and analysedduring the current study are not publicly available due Information involving trade secrets, personal privacy, and other disclosure that may harm the legitimate rights and interests of third parties shall not be disclosed. The original data belongs to trade secrets and therefore should not be disclosed. But are available from the corresponding author on reasonable request.
